# An Economical Method of Auricular Splinting in Management of Auricular Pseudocyst

**Published:** 2018-05

**Authors:** Karthik Rao, Mohan Jagade, Vitthal Kale, Dev Kumar, Amol Hekare

**Affiliations:** Grant Medical College and Sir JJ Group of Hospitals, Mumbai, Maharashtra, India

**Keywords:** Auricular, Splinting, Pseudocyst, Management

## Abstract

**BACKGROUND:**

Pseudocyst of the auricle is a common benign disease. Many treatment modalities have been described for this benign condition ranging from simple aspiration to complex cutaneous surgeries involving skin de-roofing and debridement with diamond burr. the aim of treatment is to successfully resolve the seroma without damaging the underlying healthy cartilage, thus maintaining the normal contour of the auricle, and to prevent its recurrence.

**METHODS:**

In this study we describe incision and drainage of the pseudocyst with auricular splinting.

**RESULTS:**

Resolution was seen in 100.00 %, skin discolouration in 33.33%, skin thickening in 29.63% and deformity in 25.93% of the patients.

**CONCLUSION:**

The use of corrugated drain sheet splint is an ingenious method of aural pseudocyst management. This method is simple and can be performed by even less experienced surgeons and highly economical which prevents the recurrence and maintains the auricular aesthetics.

## INTRODUCTION

Pseudocyst of the auricle is a common benign disease more predominant in the Chinese population,^1^ characterized by spontaneous, asymptomatic swelling over the anterior aspect of the auricle. Many treatment modalities have been described for this benign condition ranging from simple aspiration to complex cutaneous surgeries involving skin de-roofing and debridement with diamond burr. Multiple intralesional therapies have also been tried with corticosteroids, 50% TCA and Bleomycin with displeasing results. Thus the aim of treatment is to successfully resolve the seroma without damaging the underlying healthy cartilage, thus maintaining the normal contour of the auricle, and to prevent its recurrence.

## MATERIALS AND METHODS

The inclusion criteria were patients of auricular seroma visiting the OPD of ENT and Head – Neck surgery, Grant Government Medical College, Mumbai, India. The exclusion criteria were any coexistent disease of the pinna, i.e. abscess, dermatological conditions, congenital anomalies, and any coexistent disease of the external ear or middle ear. An informed valid and written consent was taken from the patients undergoing the procedure. Under strict aseptic conditions, the patients were given aural block using 26G needle and 2% lignocaine and 1: 100,000 adrenaline. 

A cotton ball was placed in the outer EAC. Once the patient was adequately anesthetised, an incision parallel to the crus of antihelix was taken. The seroma was drained through the incision site by applying pressure over the skin, the blunt end of the Freer’s speculum was passed through the site of incision to break the septations if existed. A betadine wash was given through the wound. After adequate drainage of the seroma, a corrugated rubber sheet was fashioned into the shape of affected pinna. In anterior splint, triangular cut was made into the anterior end of the sheet facilitating the drainage, if there is reaccumilation.

**Fig. 1:**
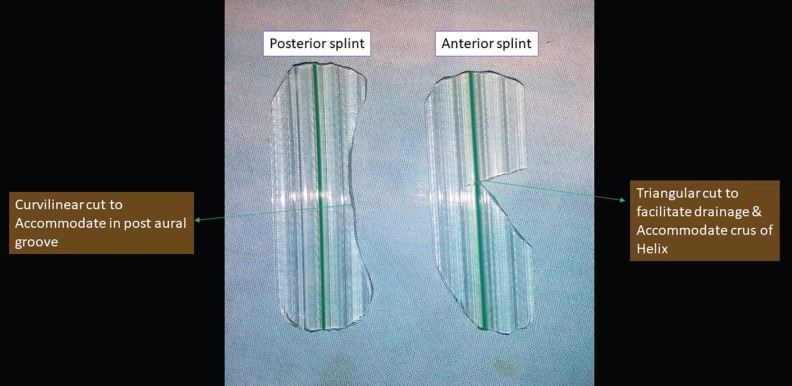
The corrugated drain sheet fashioned into anterior and posterior splints. The anterior splint having triangular cut to facilitate drainage and accommodate crus of Helix; the posterior splint having curvilinear cut to accommodate in post-aural groove

**Fig. 2:**
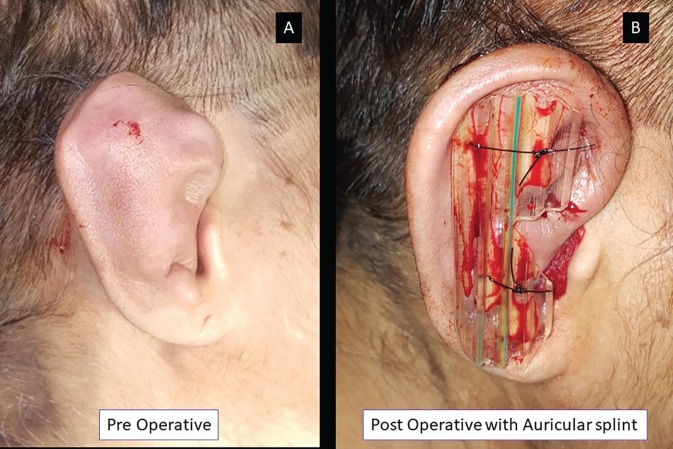
The pre-operative photograph (panel A) depicting extensive chronic pseudocyst of the right auricle following trauma. Post-operative photograph with aural splint is shown in panel B

In posterior splint, curvilinear cut was made to accommodate into the post aural groove (Figure 1). These splints were used to sandwich the affected pinna from anterio-posteriorly by using 3-0 Ethilon reverse cutting. The wound was bathed in mupirocin ointment. Post-procedure, the wound with the splint were kept open (Figure 2). Patients received oral antibiotics and an analgesic and were reviewed on every alternate day for two weeks. Patients were educated to avoid trauma or soakage of the dressing and were advised to review with us immediately, if they experienced excessive pain, diffuse redness, discharge or fever. The patients were examined for any reaccumilation, discoloration of the pinna, deformity, thickening, or signs of perichondritis. After removal of the splint, the patients were followed up for 6 months. 

## RESULTS

**Fig. 3:**
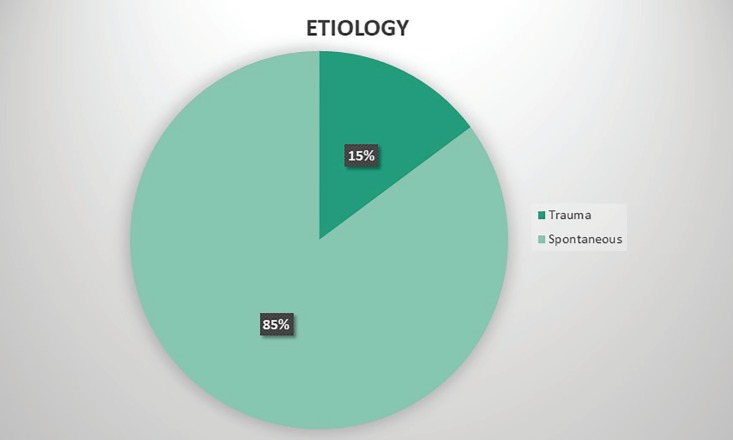
Pie chart depicting the aetiology of pseudocyst

**Fig. 4:**
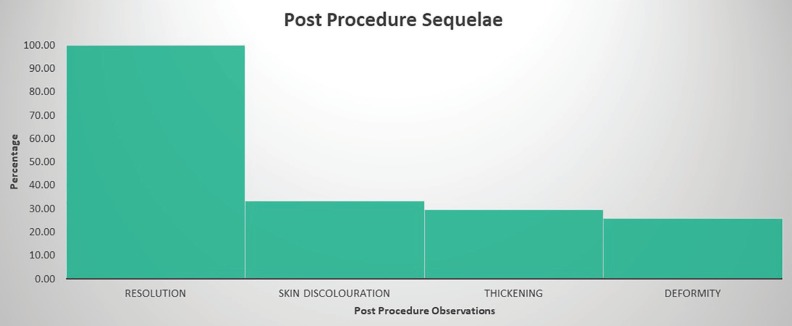
Bar graph depicting the post-operative observations during follow up

**Fig. 5:**
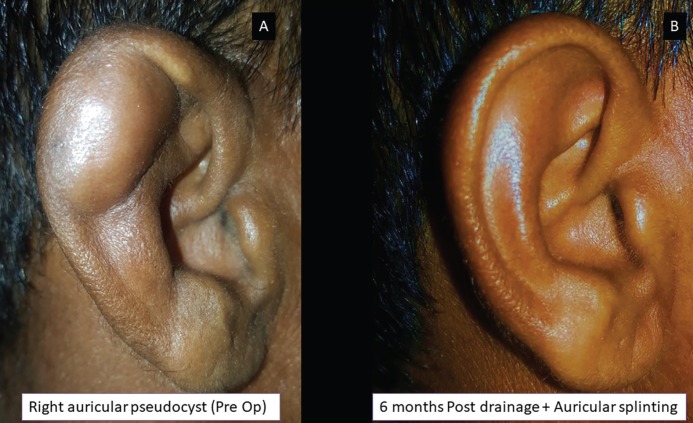
Localized pseudocyst of right ear (panel A) and complete resolution seen during the 6^th^ month follow up (panel B)

A total of 27 patients were taken into the study, with 24 males and 3 females. Median age group was 41.19 years, 20 patients had right sided lesion, 7 had left sided lesion, none of the patients had bilateral lesions. Trauma was predisposing factor for 4 patients (14.81%) and significant etiological factor was not identifiable in other patients in 23 patients (85.19%) (Figure 3). Following the aspiration contour dressing and splint application (Figure 4), in all 27 patients (100%) there was complete resolution of swelling (Figure 5). No patients have reaccumilation of fluid in the ear. The patients were asked to continue with the antibiotics and analgesics orally along topical mupirocin. 

**Fig. 6:**
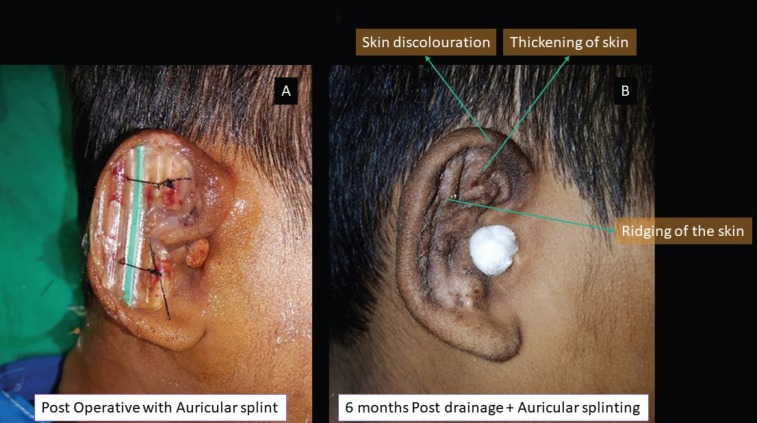
Panel A showing the post-operative photograph and Panel B depicting the skin discolouration, thickening of skin and ridging seen during the 6^th^ month follow up

**Fig. 7:**
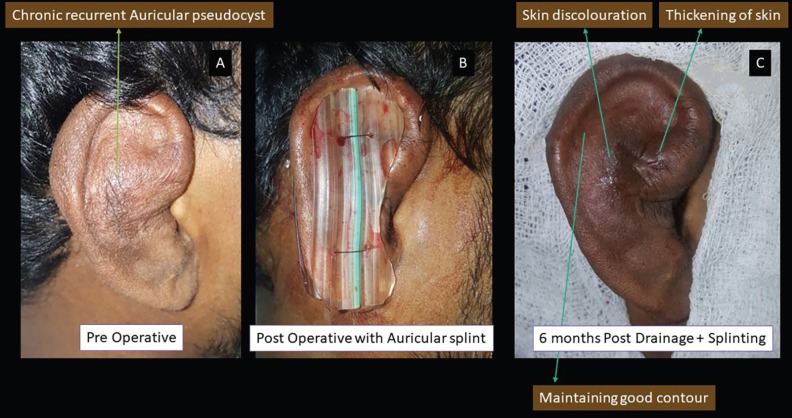
Panel A showing pre-operative photograph of chronic recurrent auricular pseudocyst, Panel B showing immediate post-operative photograph and panel C showing 6^th^ monthly follow up photograph depicting maintenance of good aural contour with mild skin discolouration and thickening of skin

Discolouration of the pinna (Figure 6 and 7) was noted at 2 weeks following the splint removal in 9 patients (33.33%). Skin thickening over the pinna was seen in 8 patients (29.63%). The skin thickening and discolouration were managed with topical emollients which resolved in 2 weeks. Seven patients (25.93%) had residual deformities of the pinna such as loss of contour and ridging of skin corresponding to the corrugated rubber sheet (Figure 6). The skin ridging resolved in 2-3 weeks without any further therapy. None of the patients had any major complications like perichondritis or aural abscess.

## DISCUSSION

Auricular seroma of the auricle were initially reported in Chinese men.^1^ Auricular seroma, also known as pseudocyst of the pinna, which is typically unilateral, asymptomatic and cystic swellings predominantly in the right ear. Male preponderance is observed. in our study, the age of the affected patients ranged from 16 to 62 years with 24 males (87%) and 3 females (13%). The literature suggests that the age group 15-85 are affected more.^2^ Mostly, the cause for the pseudocyst cannot be identified. 

The differential diagnosis includes subperichondral hematoma caused by accumulation of blood secondary to trauma, relapsing polychondritis, and cellulitis. Pseudocyst commonly occur as a post trauma sequela. Multiple hypothesis has been put forth explaining the seroma formation such as lysosomal degradation of cartilage which is a consequence of a trauma. There have been postulates which suggests the gaps in the auricular cartilage, these gaps tend to open the tissue planes when subjected to repeated minor trauma or mechanical stress, forming a pseudocyst.^1^^,^^3^

In our case study, only 4 patients (14.81%) had history of trauma. Several methods of treating auricular pseudocysts are described in the literature. This may be due to the high recurrence rates reported with aspiration alone. When pseudocysts do recur it is typically within several weeks. So, the ideally aim of the treatment is successful resolution of the seroma without damage to the healthy cartilage, thus maintaining the normal contour of the auricle, and to prevent its recurrence. Many treatment modalities have been reported in the literature include simple aspiration, intralesional injection of corticosteroids, aspiration in combination with bolstered pressure sutures, aspiration with cruciate incision, and application of POP cast following drainage. 

Chang *et al.* described a technique under local anaesthesia consisting of de-roofing the anterior wall of the Pseudocyst after exposure through a skin incision along the antihelical line. The anterior leaflet of cartilage was resected along the circumference of the swelling and the posterior leaflet was curetted to remove debris and granulation tissue. Resuturing of the skin was performed to return it to its original position and a compression dressing was then applied for two days.^4^ However, the invasive treatment modalities carry the risk of perichondritis complicated by formation of a floppy ear or cauliflower deformity and may be followed by recurrences.^5^^,^^6^

The main disadvantage associated with intralesional steroid administration such as skin pigmentation changes, and cartilage atrophy. Other methods were (i) Aspiration with pressure dressing using cotton–recurrence rate of 60 to 96.55%,^7^ (ii) Incision and drainage with buttoning-recurrence rate of 38% to 40%,^7^ (iii) Simple aspiration followed by intralesional steroid injection followed by pressure dressing–recurrence rate of 43%, (iv) In needle aspiration with intralesional steroid injection and contour dressing - recurrence rate of 85.41%, (v) Surgical de-roofing of the pseudocyst–recurrence rate of 10%,^4^^,^^8^ and (vi) Surgical de-roofing with buttoning, no reported recurrence.

Certain authors have been aggressive in their approach by excising of the anterior wall of the pseudocyst followed by abrasion of granulation tissue on the inner wall with a diamond burr, followed by a bolster dressing and two weeks postoperatively the patient had an area of exposed cartilage that required a full thickness post auricular skin graft.^9^ In our study following the aspiration, contour dressing and splint application, in all 27 patients (100%), there was complete resolution of swelling. No patient had reaccumilation of fluid in the ear. 

The method used in the study is of simple needle aspiration and local pressure application and ear splinting is simple can be performed by even an entry level surgical trainee, minimally invasive, economical and provides adequate aesthetic outcomes. The appropriate strength and thickness of the corrugated sheet make it convenient for the surgeons to trim and shape it to the lesion size and to provide equal amounts of pressure on both sides of the lesion. Compression of corrugated sheet is firm and provides an equal pressure on both sides of the pinna, leading to the adhesions of skin and dorsal cartilage, elimination of the dead cavity, and the inhibition of further effusion.

The surface of the drain sheet is not smooth and has ridges. This has led to ridging over the skin following the splint removal. Ridging resolved in 2-3 weeks. Discolouration of the pinna was noted at 2 weeks following the splint removal in 9 patients (33.33%). Skin thickening over the pinna was seen in 8 patients (29.63%). The skin thickening and discolouration were managed with topical emollients which resolved in 2 weeks.

The potential risk associated with compressive techniques, such as a Aqualplast compressive ear prosthesis for pressure application, may include pressure necrosis if the device is too tight.^10^ Corrugated drain sheet usage as splint gives optimum and uniform pressure over the auricle preventing reaccumilation of seroma and the risk of pressure necrosis and perichondritis. The skin changes could be explained by the non-porosity of the rubber sheet. The use of corrugated drain sheet splint is an ingenious method of aural pseudocyst management. This method is simple can be performed by even less experienced surgeons and highly economical which prevents the recurrence maintains the auricular aesthetics.

## CONFLICT OF INTEREST

The authors declare no conflict of interest.
